# Age-Related Stress in Individuals with a Substance Use History

**DOI:** 10.1093/geroni/igaf122.4065

**Published:** 2025-12-31

**Authors:** Suruchi Ghaiye, Ge Wang, Daniel Li, Runze Ma, Wei Li

**Affiliations:** University of Alabama at Birmingham, Birmingham, Alabama, United States; Huazhong University of Science and Technology, WuHan, Hubei, China (People’s Republic); SUNY, New York, New York, United States; University of Alabama at Birmingham, Birmingham, Alabama, United States; UAB, Birmingham, Alabama, United States

## Abstract

Substance use recovery is a multifaceted process influenced by psychological, physiological, and social factors. Stress remains a critical barrier to sustained recovery, particularly among elderly individuals who may face compounded challenges due to age-related vulnerabilities. Understanding age-related stress in recovery populations can inform targeted interventions and improve program efficacy. Demographic information and vital signs were collected from participants aged from 20 to 80 years old. Stress was assessed using a standardized questionnaire. Correlation analyses was done between demographic factors or vital signs and stress scores using the SPSS. p < 0.05 was considered significant. Age was positively correlated with systolic blood pressure (r = 0.235, p = 0.03) and diastolic blood pressure (r = 0.258, p = 0.017), indicating higher blood pressure levels in older participants. Conversely, heart rate showed a negative correlation with age (r = −0.216, p = 0.046). Psychological stress negatively correlated with age and older individuals reported lower levels of perceived stress as indicated by the following negative correlations: 1. feeling nervous or stressed (r=-0.369, p < 0.001); 2. feeling unable to control important things (r=-0.256, p = 0.017); 3. perceived helplessness (r=-0.286, p = 0.008); Total stress score (r=-0.25, p = 0.02). In conclusion, age is a significant factor influencing stress in individual enrolled in a local substance abuse recovery program. These findings underscore the need for age-sensitive approaches in substance use recovery services and highlight the importance of continuous psycho-social support.

